# A Simple and Efficient Approach to Elucidate Genomic Contribution of Transcripts to a Target Gene in Polyploids: The Case of Hexaploid Wheat (*Triticum aestivum* L.)

**DOI:** 10.3389/fpls.2016.01597

**Published:** 2016-10-27

**Authors:** Vijaya R. Chitnis, Tran-Nguyen Nguyen, Belay T. Ayele

**Affiliations:** Department of Plant Science, University of ManitobaWinnipeg, MB, Canada

**Keywords:** polyploids, wheat, genome specificity, gene expression, homeologs

## Abstract

Common wheat (*Triticum aestivum* L.) is one of the most economically important crops in the world, however, gene functional studies in this crop have been lagging mainly due to the complexity of its polyploid genome, which is derived through two rounds of intergeneric hybridization events that led to the presence of six copies for each gene. Elucidating the transcript contribution of each genome to the total expression of a target gene in polyploids such as hexaploid wheat has a paramount significance for direct discovery of genes and the associated molecular mechanisms controlling traits of agronomic importance. A polymerase chain reaction approach that involved primers amplifying DNA fragments unique to each homeolog of a target gene and quantitation of the intensity of the resulting fragment bands were able to successfully determine the genomic transcript contributions as a percentage of target gene's total expression in hexaploid wheat. Our results showed that the genomic contributions of transcripts to a target gene vary with genotype and tissue type, suggesting the distinct role of each homeolog in regulating the trait associated with the target gene. The approach described in this study is an effective and economical method to elucidate the genomic transcript contribution to the total expression of individual target genes in hexaploid wheat. It can also be applied to determine the transcript contribution of each genome towards the collective expression of a target gene in other economically important polypoid crop species.

## Introduction

Common wheat (*Triticum aestivum* L.) is one of the most important crops world-wide, contributing to ~95% of the global wheat production while durum wheat accounts for most of the remaining 5% of wheat production (Dubcovsky and Dvorak, 2007). Common wheat is allopolyploid characterized by a complex genome originated from three diploid species through two rounds of intergeneric hybridization events (He et al., 2003). The first round of intergeneric hybridization involved two diploid species, *Triticum urartu* as A genome progenitor and a yet-undiscovered or extinct *Aegilops* species closely related to *Aegilops Speltoides* as B genome progenitor (Feldman and Levy, 2012), and produced a tetraploid durum wheat (*Triticum turgidum* ssp. *durum*) with genome composition of AABB (2*n* = 28). The second round of intergeneric hybridization and polyploidization involved *T*. *turgidum* (AABB), and *Aegilops tauschii*, as diploid progenitor of the D genome (Feldman and Levy, 2012) and produced the hexaploid common wheat (*T*. *aestivum*) with genome composition of AABBDD (2*n* = 42). Each subgenome in both *T*. *turgidum* and *T*. *aestivum* consists of seven pairs of homeologs chromosomes, as a result each gene has four and six copies, respectively. This polyploidization/duplication leads to divergence in gene expression that may result in gain in new expression state, partitioning of their ancestral function, or complete loss of their expression state and pseudogenization (Moghe and Shiu, 2014). For example, majority of duplicated wheat genes that encode enzymes are active (Hart, 1987), and this might cause an extra genetic dose of such genes. However, an increase in genetic dosage is not necessarily associated with beneficial effects, rather it may cause deleterious effects through inducing functional redundancy or unbalanced gene systems (Feldman and Levy, 2012).

One of the main goal of functional genomics studies in crops is to identify genes of agronomic importance through forward or reverse genetic approaches. Wheat genomic resources that are made available through next-generation sequencing of the whole genome (Brenchley et al., 2012) and those being generated through the on-going chromosome-based wheat genome sequencing by the International Wheat Genome Sequencing Consortium are playing important roles in accelerating the identification of novel functional genes. Gene expression analysis is one of the fundamental steps in functional genomics studies as it provides important insights into the physiological function of the target gene(s); it can be performed at genome-wide/large scale or target gene level. Although genome-wide/large scale gene expression analysis has the advantage of measuring the expression level of a large number of genes at the same time, it is expensive and resource intensive. For direct gene discovery, examining the spatial and temporal expression patterns of the targeted gene is an effective and economical approach. However, in polyploidy species such as allotetraploid and allohexaploid wheat, each target gene has four or six copies, respectively, which contribute transcripts to its total expression. Elucidating the contribution of each homeolog or genome to the total expression of a given target gene has a paramount significance for detailed dissection of the molecular mechanisms underlying the regulation of the associated trait of economic importance. Here, we report a strategy that is able to determine genomic contributions to the total expression of individual target genes in hexaploid wheat (Deol et al., 2013; Mukherjee et al., 2015; Son et al., 2016) using the homeologs of two recently reported genes encoding enzymes that catalyze specific steps in the metabolic pathway of abscisic acid (ABA) (Chono et al., 2013; Son et al., 2016), a plant hormone implicated in regulating several traits of agronomic importance including seed dormancy (Gao and Ayele, 2014).

## Materials and methods

### Plant materials and growth conditions

Two genotypes of hexaploid wheat, AC Domain and RL4452, were grown in a greenhouse at 18–22⋅C/14–18⋅C (day/night) under a 16/8 h photoperiod; the seeds for these genotypes were kindly provided by Dr. Gavin Humphreys of Agriculture and Agri-Food Canada-Cereal Research Center (Winnipeg, Manitoba, Canada). The flag leaf, peduncle, and internode tissues were harvested from each genotype at ~14 weeks after planting in liquid N_2_, and tissues were then stored at −80⋅C until further use. Two to three independent biological replicates of each tissue were collected at each stage.

### RNA extraction and cDNA synthesis

Total RNA from flag leaf tissue was extracted as described before (Nguyen et al., 2016) while the total RNA samples from the peduncle and internode tissues were extracted using the GeneJET Plant RNA Purification Mini Kit (ThermoFisher, Waltham, MA, USA) following the manufacturer's instruction. After determination of the purity and integrity of the total RNA with an Epoch Microplate Spectrophotometer (Biotek, Winooski, VT, USA) and agarose gel electrophoresis, respectively, the total RNA samples were treated with DNase (Ambion, Austin, TX, USA) to eliminate any genomic DNA contamination. The DNase treated total RNA samples were then subjected to cDNA synthesis using iScript Reverse Transcription Supermix Kit (Bio-Rad) according to the manufacturer's protocol. The cDNA sample was diluted 20x and then stored in −80⋅C until further use.

### Primers and real time qPCR assay

This study involved the use of the three homeologs of ABA biosynthetic [*TaNCED2* (*TaNCED2A*, GenBank ID: KX711890; *TaNCED2B*, GenBank ID: KX711891; *TaNCED2B*, GenBank ID: KX711892; Son et al., 2016)] and ABA catabolic [*TaCYP707A1* (*TaCYP707A1A*, GenBank ID: AB714577; *TaCYP707A1B*, GenBank ID: AB714578; *TaCYP707A1D*, GenBank ID: AB714579; Chono et al., 2013)] genes of hexaploid wheat as target genes. The forward and reverse primer sequences that can amplify fragments conserved across all three homeologs of each target gene and thereby detect their collective expression are described previously (Son et al., 2016; Table 1). The wheat β*-actin* (*Ta*β*-actin*) gene was used as reference gene for expression analysis of the target genes and it was amplified using primers described before (Son et al., 2016; Table 1). The real time qPCR assays for the target and reference genes were performed as described before (Nguyen et al., 2016). Briefly, each qPCR reaction consists of 5 μl of dilute cDNA, 10 μl of SsoFast EvaGreen Supermix (Bio-Rad), 1.2 μl of 5 μM forward primer (300 nM final concentration), 1.2 μL of 5 μM reverse primer (300 nM final concentration), and 2.6 μL diethylpyrocarbonate treated water. The reaction mix was subjected to the following PCR condition using the CFX96 Real-Time PCR system (Bio-Rad): DNA polymerase activation at 95⋅C for 5 min followed by 40 cycles of denaturation at 95⋅C for 15 s, annealing at 60⋅C for 30 s and extension at 72⋅C for 30 s. Transcript levels of the target genes were expressed after normalization with β*-actin* according to Livak and Schmittgen (2001).

**Table 1:** **Primer sequences used in the determination of the total expression (qPCR) and genomic transcript contribution for *TaNCED2* and *TaCYP707A1***.

**Gene**	**Primer sequences**		**Amplicon size (bp)**		
	**FW (5′–3′)**	**R (5′–3′)**	
**qPCR (TOTAL EXPRESSION)**					
*TaNCED2*	CGCTTGCCGCCTCCACGTTT	AGGCTCCCTCTGGCGACTTCC	80		
*TaCYP707A1*	GCCAGGAAGCGGAACAAG	AAGAGGTGCGCCTGAGTA	119		
*Taβ-actin*	GCTGGAAGGTGCTGAGGGA	GCATCGCCGACAGGATGAG	138		
**Gene**	**Primer sequences**		**Amplicon size (genome specific bp)**		
	**FW (5′–3′)**	**R (5′–3′)**	**A**	**B**	**D**
**PCR (GENOMIC TRANSCRIPT CONTRIBUTION)**					
*TaNCED2*	GTCTCCTCCATACAGCGGC	GCTTCTTCTCCTGCCTCTCC	193	134	205
*TaCYP707A1*	ATGACCTTCACCCGCAAGG	CCTTTGGGAACGAGGAGGA	56	72	87

### Primers and PCR assay

The forward and reverse primers spanning regions that are polymorphic across the three homeologs of each target gene (*TaNCED2* and *TaCYP707A1*) and thereby amplify fragments unique to each homeolog of the target genes are described previously (Son et al., 2016), and these primers were used to perform the PCR assays. The PCR reactions for *TaNCED2* consists of 5 μl of cDNA (50 ng), 5 μl of 5x high GC enhancer, 5 μl of 5x Q5 buffer, 0.5 μl of 10 mM dNTPs, 1 μl of forward primer (5 mM), 0.5 μl reverse primer (5 mM), 0.25 μl of Q5 high fidelity DNA polymerase (2000 U; New England BioLabs, Ipswich, MA, USA), and nuclease-free water to make a total reaction volume of 25 μl. The reaction mixtures were then subjected to touchdown PCR conditions: DNA polymerase activation at 98⋅C for 3 min followed by 5 cycles of three steps: denaturation at 98⋅C for 10 s, annealing at 61⋅C for 30 s decreasing 0.5⋅C per cycle to 58.5⋅C), and extension at 72⋅C for 30 s followed by 35 additional cycles of three steps: denaturation at 98⋅C for 10 s, annealing at 58⋅C, and extension at 72⋅C for 30 s. Final extension was performed at 72⋅C for 2 min. The entire volume of the PCR product was used for running polyacrylamide gel. The PCR assays for *TaCYP707A1* consist of 5 μl cDNA (50 ng), 2 μl of 10x dream Taq buffer (containing 20 mM MgCl_2_, ThermoFisher), 0.5 μl of 10 mM dNTPs, 0.5 μl forward primer (5 mM), 0.5 μl reverse primer (5 mM), 0.12 μl of dream Taq DNA polymerase (500 U), and nuclease-free water to make a total reaction volume of 20 μl. The reaction mixtures were then subjected to the following thermocycling conditions: DNA polymerase activation at 95⋅C for 3 min followed by 40 cycles of denaturation at 95⋅C for 20 s, annealing at 55⋅C for 30 s and extension at 72⋅C for 25 s, and final extension at 72⋅C for 5 min.

### Separation of genome specific PCR fragments

The genome specific PCR fragments of *TaNCED2* were separated vertically on 15% PAGE while that of *TaCYP707A1* were separated on 10% PAGE. The 15% PAGE was prepared by mixing 15 ml of 29% Acrylamide plus 1% N, N′-metheylenebisacrylamide with 11.77 ml of water, 3 ml of 10X Tris/Borate/EDTA (TBE), and 0.22 ml of 10% Ammonium persulfate (APS) and 12 μl of N,N,N′,N′-tetramethylethylene diamine (TEMED). The 10% PAGE was prepared as described above except that 10 ml of 29% Acrylamide plus 1% N, N′-metheylenebisacrylamide and 16.77 ml of water were used. The PAGE for *TaNCED2* was run at 150 V for 22 h while that for *TaCYP707A1* was run at 100 V for 10 h. The PAGE was then stained in ethidium bromide solution (0.5 μg/ml) for 20–30 min at room temperature before visualization using the Gel Doc XR system (Bio-Rad).

### Quantitation of band intensity

The area, volume, and intensity of each band corresponding to expected size of a given amplicon were analyzed using Quantity One Software (Bio-Rad) and the global background subtraction method. The intensities of the band volumes were adjusted by subtracting the intensity of the background volume. The volume of the bands representing each specific PCR fragments was defined by creating a uniform rectangular box around each band to be quantified, and a rectangular box of the same size was also created in the region of the gel picture with no band to be considered as a background.

### Genomic contribution of transcripts

The genomic transcript contribution for the target genes, *TaNCED2* and *TaCYP707A1*, was expressed as a percentage of their total expression, which is calculated as follows: (adjusted intensity inside the volume of a band corresponding a specific homeolog/the sum of adjusted intensities inside the volumes of bands derived from the three homeologs of a target gene) ^*^ 100.

## Results and discussion

**Figure 1:**
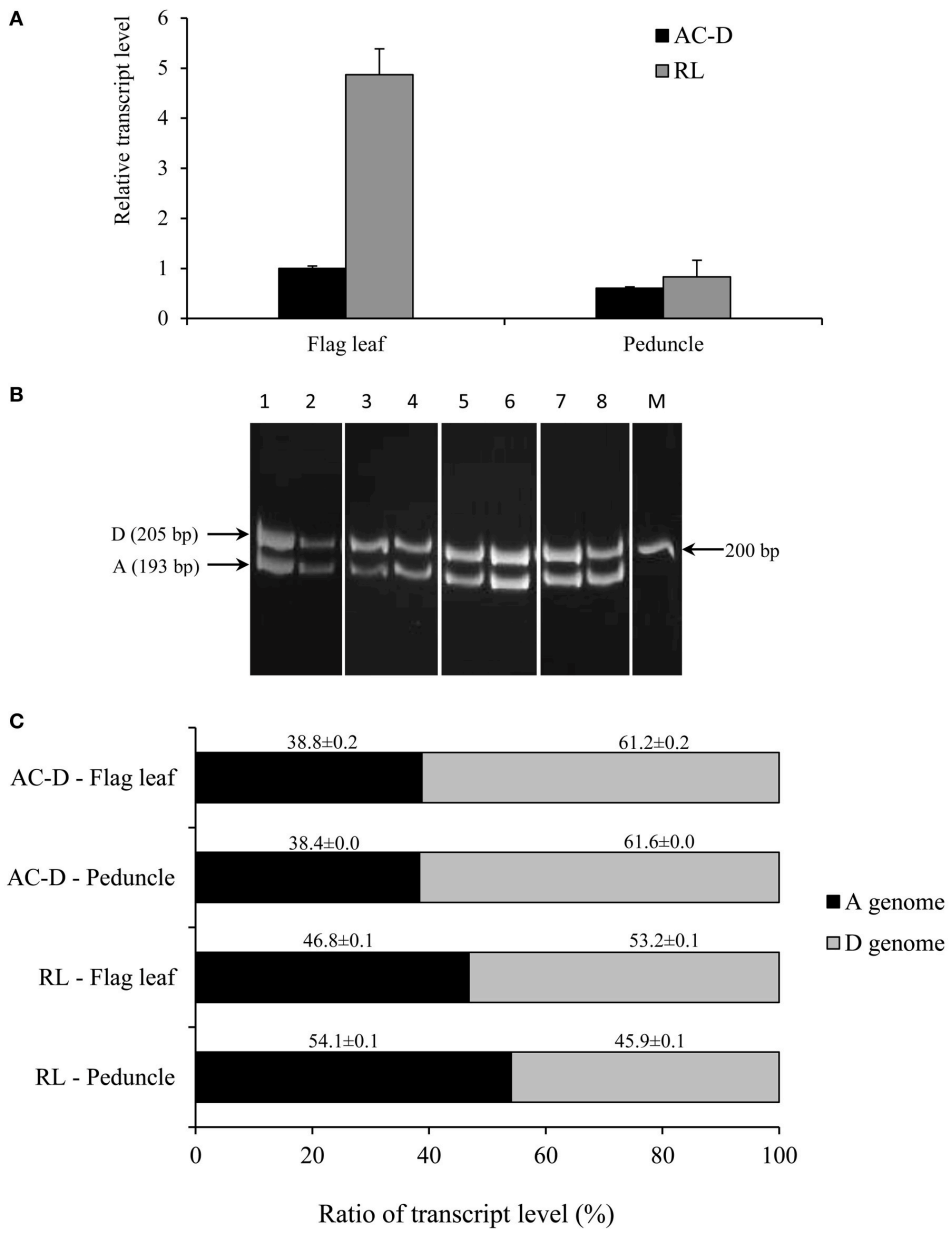
**Total expression and genomic transcript contribution for *TaNCED2* in the flag leaf and peduncle tissues of hexaploid wheat genotypes AC Domain (AC-D) and RL4452 (RL)**. Relative transcript abundance of *TaNCED2* in the tissues **(A)**. Transcript levels of *TaNCED2* were determined using β-actin as a reference gene and then expressed relative to that of flag leaf in AC-D, which was set arbitrarily to a value of 1. PCR products of cDNA samples representing DNA fragments unique to each homeolog of *TaNCED2* **(B)**. Transcript contribution of each genome as percentage to the total expression of *TaNCED2* **(C)**. Data are means ± SE, *n* = 2. M, Marker lane.

**Figure 2:**
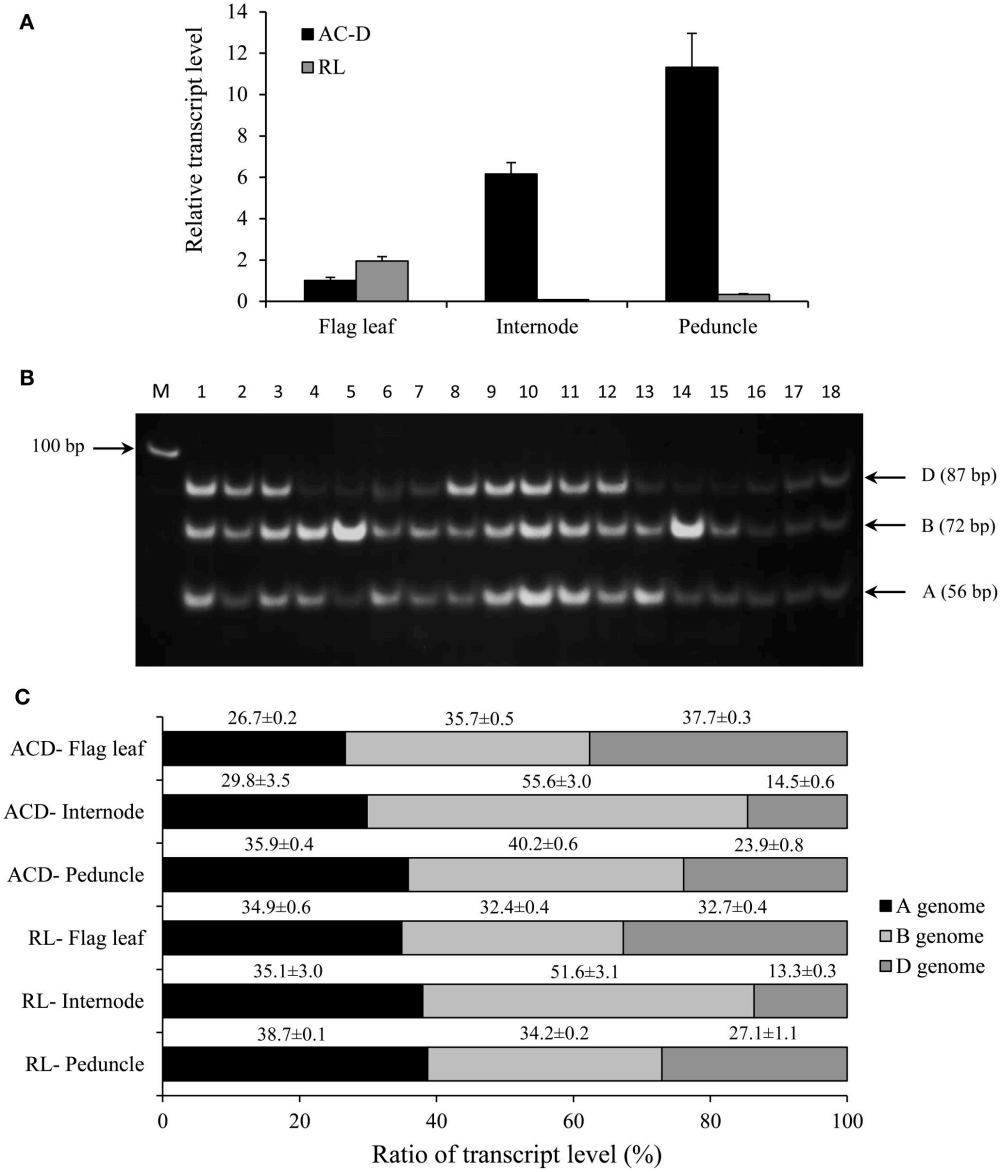
**Total expression and genomic transcript contribution for *TaCYP707A1* in the flag leaf, internode and peduncle tissues of hexaploid wheat genotypes AC Domain (AC-D) and RL4452 (RL)**. Relative transcript abundance of *TaCYP707A1* in the tissues **(A)**. Transcript levels of *TaCYP707A1* were determined using β-actin as a reference gene and then expressed relative to that of flag leaf in AC-D, which was set arbitrarily to a value of 1. PCR products representing DNA fragments unique to each homeolog *TaCYP707A1* **(B)**. Transcript contribution of each genome as percentage to the total expression of *TaCYP707A1* **(C)**. Data are means ± SE, *n* = 3. M, Marker lane.

The full length cDNA sequences of the three homeologs for the target genes of hexaploid wheat, the ABA biosynthetic gene, *9-cis-epoxycarotenoid dioxygenases* (*NCED*) (*TaNCED2*; *TaNCED2A, TaNCED2B*, and *TaNCED2D*), and the ABA catabolic gene, *cytochrome P450 monooxygenase 707A1* (*CYP707A1*) (*TaCYP707A1*; *TaCYP707A1A, TaCYP707A1B*, and *TaCYP707A1D*), were identified as described previously (Chono et al., 2013; Son et al., 2016). The total expression of the *TaNCED2* and *TaCYP707A1* in the flag leaf, peduncle, and internode tissues of two hexaploid wheat genotypes used as experimental plant materials in this study, AC Domain and RL4452, was determined using real time quantitative polymerase chain reaction (qPCR) (Figures 1A, 2A), and gene specific primers designed from the coding regions conserved across the three homeologs of each target gene (Table 1).

**Figure 3:**
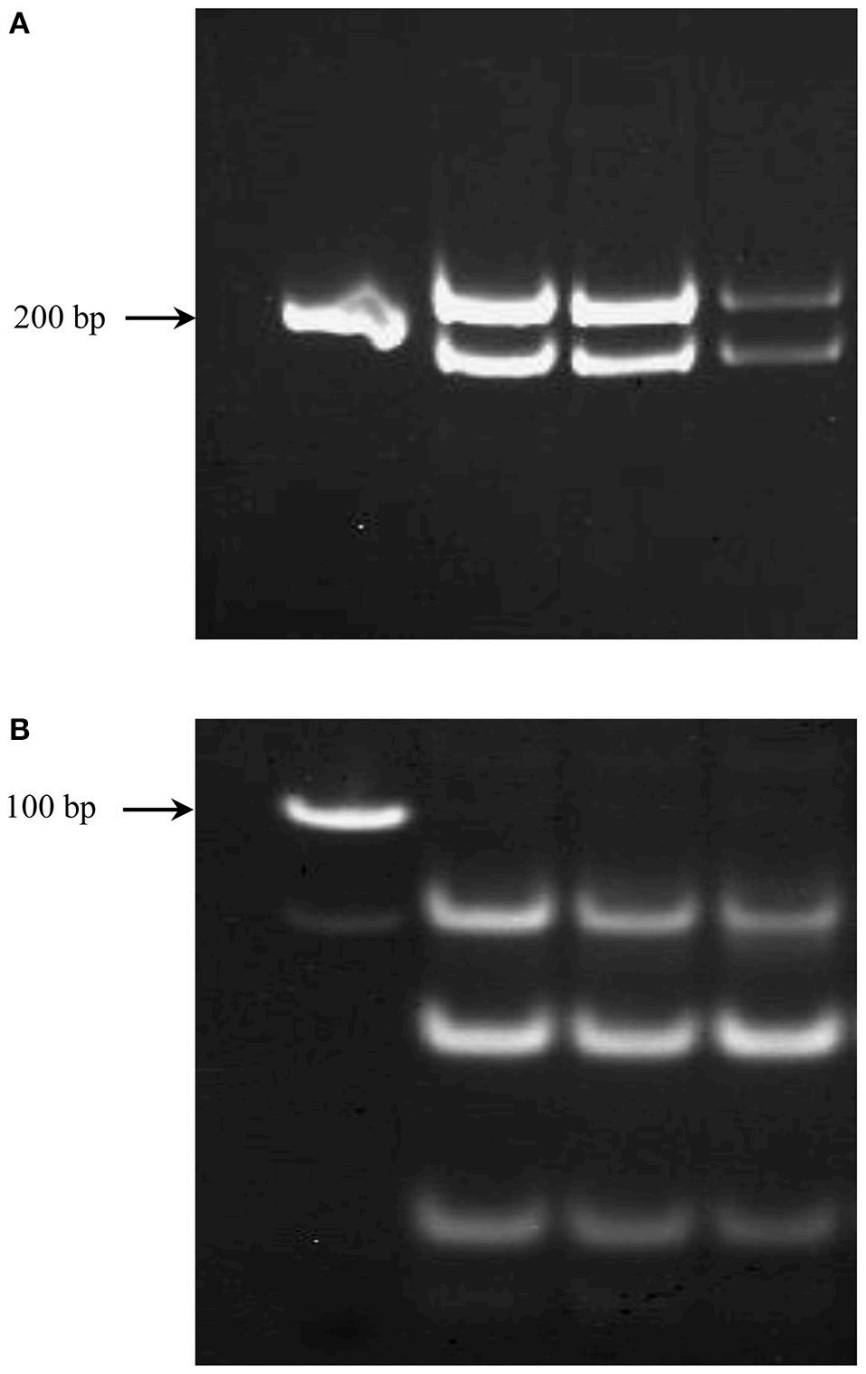
**PCR products from genomic DNA samples of different concentrations representing fragments unique to each homeolog of *TaNCED2* (A) and *TaCYP707A1* (B)**.

The genomic contributions to the total expression of *TaNCED2* and *TaCYP707A1* were, however, analyzed using specific primers that span a region that is polymorphic due to size differences among the three homeologs caused by insertion/deletion events. To this end, the full length cDNA sequences of *TaNCED2A, TaNCED2B*, and *TaNCED2D* were aligned, and the alignment revealed the presence of a polymorphic region in the coding DNA sequences of the three homeologs (Son et al., 2016). For *TaCYP707A1*, a region that is polymorphic among the *TaCYP707A1A, TaCYP707A1B*, and *TaCYP707A1D* homeologs was rather found in the 3′ untranslated regions (Son et al., 2016). The gene specific forward and reverse primers spanning the polymorphic regions of *TaNCED2* and *TaCYP707A1*, which are described previously (Son et al., 2016; Table 1), were used to amplify the three different DNA fragments unique to each homeolog of the two target genes from cDNA samples derived from the flag leaf, peduncle, and internode tissues of the two hexaploid wheat genotypes. The forward and reverse primers of *TaNCED2* were designed to amplify three different amplicons specific to *TaNCED2A* (193 bp), *TaNCED2B* (134 bp), and *TaNCED2D* (205 bp) while the primers for *TaCYP707A1* were designed to amplify three different amplicons specific to *TaCYP707A1A* (56 bp), *TaCYP707A1B* (72 bp), and *TaCYP707A1D* (87 bp). Separation of the *TaNCED2* PCR products vertically using a 15% polyacrylamide gel electrophoresis (PAGE; acrylamide: bisacrylamide ratio of 29:1) revealed the presence of only two fragments corresponding to the amplicons of *TaNCED2A* and *TaNCED2D* homeologs (Figure 1B). The absence of transcripts from *TaNCED2B* homeolog suggests that the B genome copy of *TaNCED2* is expressed at a very low/undetectable level in the genotype we analyzed. Whereas, separation of the *TaCYP707A1* PCR products using a 10% PAGE resulted in three distinct fragments corresponding to the amplicons derived from each homeolog (Figure 2B). The area, volume, and intensity of the bands representing the amplicons of each homeolog were analyzed using Quantity One Software (Bio-Rad, Hercules, CA, USA) and the global background subtraction method (Tables 2–4). As this study is focused on investigating the relative transcript contribution of each genome to the total expression of the targeted genes, no loading control/check sample or generation of standard curve is required. Amplification of the specific amplicons of the three homeologs of each gene using the primers described above was confirmed using genomic DNA samples (Figure 3).

**Table 2:** **Area, volume, and intensity of each DNA band for *TaNCED2* homeologs in AC Domain and RL4452**.

**Tissue type**	**Sample #^a^**	**Replicates**	**Genome**	**Area**	Volume INT ^*^ mm^2^	Background vol. INT ^*^ mm^2^	Adjusted vol. INT ^*^ mm^2^	Total vol. INT ^*^ mm^2^ (A + B + D)	**% Total volume**	**Average % Total volume**
**AC DOMAIN**										
Flag leaf	1	1	A	20.20	19616.66	10572.72	9043.93	23214.93	38.96	38.8 ± 0.2
			D	20.20	24743.71	10572.72	14170.99		61.04	61.2 ± 0.2
	2	2	A	20.20	19304.38	10572.72	8731.66	22608.09	38.62	
			D	20.20	24449.14	10572.72	13876.42		61.38	
Peduncle	3	1	A	20.20	22818.63	10572.72	12245.91	31879.59	38.41	38.4 ± 0.0
			D	20.20	30206.41	10572.72	19633.68		61.59	61.6 ± 0.0
	4	2	A	20.20	22294.81	10572.72	11722.08	30590.82	38.32	
			D	20.20	29441.46	10572.72	18868.74		61.68	
**RL 4452**										
Flag leaf	5	1	A	20.20	43475.07	10572.72	32902.35	70416.69	46.73	46.8 ± 0.1
			D	20.20	48087.06	10572.72	37514.34		53.27	53.2 ± 0.1
	6	2	A	20.20	43693.83	10572.72	33121.11	70581.09	46.93	
			D	20.20	48032.70	10572.72	37459.98		53.07	
Peduncle	7	1	A	20.20	36610.33	10572.72	26037.61	48234.87	53.98	54.1 ± 0.1
			D	20.20	32769.98	10572.72	22197.26		46.02	45.9 ± 0.1
	8	2	A	20.20	35118.61	10572.72	24545.88	45297.00	54.19	
			D	20.20	31323.84	10572.72	20751.12		45.81	

a *sample # refers to the lane numbers in Figure 1B; INT, intensity*.

**Table 3:** **Area, volume, and intensity of each DNA band for *TaCYP707A1* homeologs in AC Domain**.

**Tissue type**	**Sample #^a^**	**Replicates**	**Genome**	**Area**	Volume INT ^*^ mm^2^	Background vol. INT ^*^ mm^2^	Adjusted vol. INT ^*^ mm^2^	Total vol. INT ^*^ mm^2^ (A + B + D)	**% Total volume**	**Average % Total volume**
Flag leaf	1	1	A	21.73	42716.20	21896.57	20819.63	76839.66	27.09	26.7 ± 0.2
			B	21.73	48562.00	21896.57	26665.44		34.70	35.7 ± 0.5
			D	21.73	51251.16	21896.57	29354.59		38.20	37.7 ± 0.3
	2	2	A	21.73	40493.39	21896.57	18596.82	70895.53	26.23	
			B	21.73	47602.38	21896.57	25705.81		36.26	
			D	21.73	48489.47	21896.57	26592.90		37.51	
	3	3	A	21.73	41321.25	21896.57	19424.68	72739.26	26.70	
			B	21.73	48118.52	21896.57	26221.95		36.05	
			D	21.73	48989.20	21896.57	27092.63		37.25	
Internode	4	1	A	21.73	47775.75	21896.57	25879.19	77914.03	33.22	29.8 ± 3.5
			B	21.73	63368.55	21896.57	41471.98		53.23	55.6 ± 3.0
			D	21.73	32459.44	21896.57	10562.87		13.56	14.5 ± 0.6
	5	2	A	21.73	37900.63	21896.57	16004.06	69834.36	22.92	
			B	21.73	64913.42	21896.57	43016.85		61.60	
			D	21.73	32710.01	21896.57	10813.44		15.48	
	6	3	A	21.73	46889.23	21896.57	24992.66	74829.30	33.40	
			B	21.73	60821.92	21896.57	38925.36		52.02	
			D	21.73	32807.85	21896.57	10911.28		14.58	
Peduncle	7	1	A	21.73	45013.33	21896.57	23116.76	65119.26	35.50	35.9±0.4
			B	21.73	48731.05	21896.57	26834.49		41.21	40.2±0.6
			D	21.73	37064.58	21896.57	15168.01		23.29	23.9±0.8
	8	2	A	21.73	45204.81	21896.57	23308.25	65749.74	35.45	
			B	21.73	47601.69	21896.57	25705.12		39.10	
			D	21.73	38632.94	21896.57	16736.37		25.45	
	9	3	A	21.73	45300.28	21896.57	23403.71	63776.20	36.70	
			B	21.73	47638.35	21896.57	25741.78		40.36	
			D	21.73	36527.28	21896.57	14630.71		22.94	

a *sample # refers to the lane numbers in Figure 2B; INT, intensity*.

**Table 4:** **Area, volume, and intensity of each DNA band for *TaCYP707A1* homeologs in RL4452**.

**Tissue type**	**Sample #^a^**	**Replicates**	**Genome**	**Area**	Volume INT ^*^ mm^2^	Background vol. INT ^*^ mm^2^	Adjusted vol. INT ^*^ mm^2^	Total vol. INT ^*^ mm^2^ (A + B + D)	**% Total volume**	**Average % Total volume**
Flag leaf	10	1	A	21.73	58333.80	21896.57	36437.23	100905.40	36.11	34.9 ± 0.6
			B	21.73	53770.32	21896.57	31873.75		31.59	32.4 ± 0.4
			D	21.73	54490.98	21896.57	32594.42		32.30	32.7 ± 0.4
	11	2	A	21.73	54402.43	21896.57	32505.86	94186.92	34.51	
			B	21.73	53046.20	21896.57	31149.63		33.07	
			D	21.73	52427.99	21896.57	30531.42		32.42	
	12	3	A	21.73	54346.02	21896.57	32449.45	95210.02	34.08	
			B	21.73	52769.63	21896.57	30873.06		32.43	
			D	21.73	53784.09	21896.57	31887.52		33.49	
Internode	13	1	A	21.73	41154.62	21896.57	19258.05	50579.79	38.07	35.1 ± 3.0
			B	21.73	46208.28	21896.57	24311.71		48.07	51.6 ± 3.1
			D	21.73	28906.60	21896.57	7010.03		13.86	13.3 ± 0.3
	14	2	A	21.73	39521.02	21896.57	17624.45	60442.97	29.16	
			B	21.73	56785.56	21896.57	34888.99		57.72	
			D	21.73	29826.10	21896.57	7929.53		13.12	
	15	3	A	21.73	40303.74	21896.57	18407.17	48176.45	38.21	
			B	21.73	45440.45	21896.57	23543.88		48.87	
			D	21.73	28121.97	21896.57	6225.40		12.92	
Peduncle	16	1	A	21.73	34701.65	21896.57	12805.08	31974.18	40.05	38.7 ± 0.96
			B	21.73	32935.34	21896.57	11038.77		34.52	34.2 ± 0.2
			D	21.73	30026.90	21896.57	8130.33		25.43	27.1 ± 1.1
	17	2	A	21.73	33979.56	21896.57	12082.99	32787.99	36.85	
			B	21.73	33070.19	21896.57	11173.62		34.08	
			D	21.73	31427.95	21896.57	9531.38		29.07	
	18	3	A	21.73	34199.86	21896.57	12303.30	31354.33	39.24	
			B	21.73	32564.48	21896.57	10667.91		34.02	
			D	21.73	30279.69	21896.57	8383.12		26.74	

a *sample # refers to the lane numbers in Figure 2B; INT, intensity*.

Our qPCR based gene expression analysis indicated that the total expression of *TaNCED*2 in the flag leaf is greater (over four-fold) in RL4452 than that observed in AC Domain, suggesting higher ABA biosynthetic capacity in the flag leaf of RL4452 (Figure 1A). In contrast, the total expression of *TaNCED*2 in the peduncle appeared to be similar between the two genotypes. Determination of transcript contribution of each homeolog to the total expression of *TaNCED*2 revealed that the D genome copy of *TaNCED*2 contributes the most transcripts (53–61%) in the flag leaf of both genotypes followed by the A genome copy (39–47%; Figure 1C), suggesting that *TaNCED2D* accounts for majority of the TaNCED2 activity in the flag leaf tissue. No transcript contribution was observed from the B genome copy of *TaNCED*2. Further comparison showed that contribution of the D genome is higher in AC Domain (~61%) than in RL4452 (~53%) while the A genome contributes more transcripts in RL4452 (~47%) than in AC Domain (~39%). In the peduncle, the predominance of a homeolog in transcript contribution appeared to vary mainly with genotype; the D genome copy of *TaNCED2* contributes the most transcripts in AC Domain (~62%) followed by A genome copy (~38%) while the A genome copy of *TaNCED*2 contributes the most transcripts in RL4452 (~54%) followed by the D genome copy (~46%). These results suggest that the activity of TaNCED2 in the peduncle is mediated primarily by *TaNCED2D* in AC Domain but by *TaNCED2A* in RL44523. It therefore appeared from our results that genomic contribution of transcripts to the total expression of *TaNCED*2 varies with genotype and tissue, and this may imply the distinct role of each homeolog in regulating the associated trait, in this case, the synthesis of ABA.

The total expression of *TaCYP707A1* in the flag leaf is found to be similar between the two genotypes while its total expression in the internode and peduncle is substantially higher in AC Domain than that observed in RL4452 (Figure 2A). These results might suggest the presence of enhanced ABA inactivation in the internode and peduncle tissues of AC Domain than that in RL4452. Comparison between tissues in AC Domain revealed that the expression of *TaCYP707A1* was highest in the peduncle and lowest in the flag leaf (Figure 2A), suggesting the presence of reduced ABA inactivation in the flag leaf of AC Domain relative to that occurs in the internode and peduncle tissues. In the RL4452 genotype, *TaCYP707A1* is expressed at low level across the three tissues. Analysis of transcript contribution by each genome to the total expression of *TaCYP707A1* indicated that all the three genomes exhibit a similar contribution of transcripts to the total expression of *TaCYP707A1* in the flag leaf in both genotypes, although lower contribution of the A genome copy and slightly higher contributions of the B and D genome copies are observed in the AC Domain than in the RL4452 genotype (Figure 2C). In the internode of both genotypes, genome B appeared to contribute the most transcripts followed by genome A and then by genome D, implying that *TaCYP707A1B* accounts for the majority of internode TaCYP707A1 activity. When the two genotypes are compared, the transcript contribution of B genome to the total expression of *TaCYP707A1* in the internode is higher in AC Domain (~56%) than RL4452 (~48%) while the transcript contribution of A genome is higher in RL4452 (38%) than AC Domain (~30%). The D genome appeared to have similar contribution in the internode of both genotypes. As observed for *TaNCED*2, the predominance of a genome in transcript contribution in the peduncle appeared to vary mainly with genotype in which the B genome contributes the most in AC Domain (~40%) followed by genome A (~36%) and then by genome D (~24%) while genome A contributes the most (~39%) followed by genome B (~34%) and then by genome D (~27%) in RL4452. These results imply that the activity of TaCYP707A1 in the peduncle is regulated mainly by *TaCYP707A1B* in AC Domain but by *TaCYP707A1A* in RL44523. Our results again indicate that the genomic contribution of transcripts to the total expression of *TaCYP707A1* varies with genotype and tissue, reflecting the genetic and spatial regulation of each homeolog in controlling the associated trait, in this case, inactivation of bioactive ABA.

In summary, the method described in this study involves the use of PCR assays and conventional reagents and supplies, and equipment commonly available in a basic molecular biology laboratory, making it an alternative simple and cost effective approach for determining the genomic contribution of transcripts to the total expression of a target gene in the polyploidy wheat especially when the analysis is needed in a large number of samples. Such a knowledge significantly contributes to the dissection of molecular mechanisms underlying the roles of a candidate functional gene in controlling a given trait of agronomic importance. The method can also be applied to elucidate the genomic contribution of transcripts in other economically important allopolyploid crop species including cotton, oat, and canola.

## Author contributions

BA conceived the experiments. BA, VC, and TN designed the experiments and wrote the manuscript. VC performed the experiments and analyzed the data. All authors read and approved the final manuscript.

### Conflict of interest statement

The authors declare that the research was conducted in the absence of any commercial or financial relationships that could be construed as a potential conflict of interest.

## Abbreviations

ABA: abscisic acid
APS: ammonium persulfate
CYP707A: cytochrome P450 monooxygenase 707A
NCED: 9-cis-epoxycarotenoid dioxygenase
PAGE: polyacrylamide gel electrophoresis
PCR: polymerase chain reaction
TBE: Tris/Borate/EDTA
TEMED: N, N, N′, N′-tetramethylethylene diamine.
